# Fracture by Torsion

**Published:** 1900-07-21

**Authors:** 


### FRACTURE BY TORSION.

A good deal has been said of late about the influence of torsion in the production of fracture of bones, and it has been pointed out that it is the impossibility of undoing the "twist" that is one cause at least of the difficulty of properly and completely reducing certain fractures, especially in the leg, without laying the whole thing open and dealing with the bone as a joiner would deal with the broken leg of a table. That there is much truth in this contention as to the manner in which bones break will be at once granted by those who witnessed the demonstration given by Dr. Griffiths, of Cambridge, on the occasion of the Pathological Society meeting at the Pathological Laboratories of the University of Cambridge, at the invitation of Professor Sims Woodhead. Dr. Griffiths showed the various ways in which bones break in accordance with the direction in which the breaking force may happen to be applied to them, and he pointed out how important a part was played by torsion in the production of fracture. The effect of torsion was to produce a spiral fracture, and he showed that the character of the spiral depended, among other reasons, on the amount of "bending" as well as "twisting" force which was employed. If the bone were bent as well as twisted, the spiral was shortened. What he said about fractures of the spine, which are probably almost entirely due to torsion, evidently has an important bearing upon the question how far it can ever be possible to restore such bones to their original position.

